# Photosynthesis Product Allocation and Yield in Sweet Potato in Response to Different Late-Season Irrigation Levels

**DOI:** 10.3390/plants12091780

**Published:** 2023-04-26

**Authors:** Mingjing Zhou, Yiming Sun, Shaoxia Wang, Qing Liu, Huan Li

**Affiliations:** College of Resources and Environmental Sciences, Qingdao Agricultural University, Qingdao 266109, China

**Keywords:** sweet potato, late irrigation, ^13^C allocation, economic return and yield

## Abstract

Soil water deficit is an important factor affecting the source–sink balance of sweet potato during its late-season growth, but water regulation during this period has not been well studied. Therefore, the aim of this study was to determine the appropriate irrigation level in late-season sweet potato, and the effect of irrigation level on accumulation and allocation of photosynthetic products. In this study, two yield-based field trials (2021–2022) were conducted in which five late-season irrigation levels set according to the crop evapotranspiration rate were tested (T_0_: non-irrigation, T_1_: 33% ET_c_, T_2_: 75% ET_c_, T_3_: 100% ET_c_, T_4_: 125% ET_c_). The effects of the different irrigation levels on photosynthetic physiological indexes, ^13^C transfer allocation, water use efficiency (WUE), water productivity (WP), and the yield and economic benefit of sweet potato were studied. The results showed that late-season irrigation significantly increased the total chlorophyll content and net photosynthetic rate of functional leaves, in addition to promoting the accumulation of above-ground-source organic biomass (*p* < 0.05). The rate of ^13^C allocation, maximum accumulation rate (V_max_), and average accumulation rate (V_mean_) of dry matter in storage root were significantly higher under T_2_ irrigation than under the other treatments (*p* < 0.05). This suggests that both non-irrigation (T_0_) and over-irrigation (T_4_) were not conducive to the transfer and allocation of photosynthetic products to storage roots in late-season sweet potato. However, moderate irrigation (T_2_) effectively promoted the source–sink balance, enhanced the source photosynthetic rate and stimulated the sink activity, such that more photosynthate was allocated to the storage sink. The results also showed that T_2_ irrigation treatments significantly increased yield, WUE and WP compared to T_0_ and T_4_ (*p* < 0.05), suggesting that moderate irrigation (T_2_) can significantly promote the potential of storage root production and field productivity. There was a close relationship between economic benefit and marketable sweet potato yield, and both were highest under T_2_ (*p* < 0.05), increasing by 36.1% and 59.9% compared with T_0_ over the two-year study period. In conclusion, irrigation of late-season sweet potato with 75% evapotranspiration (T_2_) can improve both the yield and production potential. Together, these results support the use of late-season water management in the production of sweet potato.

## 1. Introduction

Source and sink, and specifically their establishment, development and balance, are important determinants of the economic yield of crops [1]. In the source–sink relationship, plant water is a crucial and manageable factor [2]. Understanding the myriad plant processes and mechanisms that determine crop water requirements is a prerequisite for improving agronomic performance [3,4].

Sweet potato [*Ipomoea batatas* (L.)] is a food crop that is highly stress resistant and adaptable [5]. However, while sweet potato is moderately drought-tolerant, it is sensitive to water deficit, particularly during its early (establishment) stage of growth, which includes early vine development and storage root initiation [6]. The early stage is also vital for establishing the source–sink relationship [7]. Consequently, the water supply for sweet potato rooting and branching is often guaranteed through measures such as plastic mulching and adequate seedling watering [6]. In addition to stimulating the development and early initiation of storage roots [8], sufficient soil moisture ensures proper storage root bulking and the maintenance of leaf area during mid-season, which contributes to the development of the source–sink relationship [9]. Fortunately, summer rainfall can fully meet the water demand during this period. Regions of most areas of northern China that produce sweet potato are prone to variable soil moisture, and fluctuations in soil moisture during the autumn months may affect crop survival, performance, and final yield and quality of storage roots [10]. During the late stage of sweet potato (the “balance stage” of the source–sink relationship), however, water management is no longer carried out and there is a reliance on natural precipitation to maintain growth [7,11]. Consequently, the importance of water regulation during the late stage of sweet potato cultivation has not been well studied.

Source–sink balance is an ideal state reached in the late growth stage of sweet potato, when the photosynthetic efficiency of the source leaves and proportion of synthesized photosynthate distributed to the storage roots are highest. During the late growth period, prolonged maintenance of a balanced source–sink relationship prevents insufficient growth of source leaves, which would reduce photosynthetic efficiency, as well as their excessive growth (which would reduce the proportion of photosynthates distributed to storage roots) [12]. In sweet potato, a prolonged soil moisture deficit during the mid and late growth stages limits canopy development, and thus, photosynthetic activity, which ultimately affects storage root development, bulking, yield, and quality [13]. Jones et al. (1985) noted a larger effect of a soil moisture deficit during the storage root bulking stage of sweet potato than during the initial stage of its vegetative growth. Accordingly, producers often use drip irrigation to achieve multiple irrigation cycles and thereby effectively alleviate seasonal drought [14]. Late-season irrigation after drought has been studied in potato [15], onion [16] and tomato [17]. However, while Thomson et al. (1992) and Gajanayake et al. (2016)conducted water management and modelling studies in sweet potato, the focus in both cases was the middle growth period, such that little is known about the effects of late-season irrigation [18,19]. Excess soil moisture has detrimental effects on the growth and development of field crops [20], and reduces the storage root yield of sweet potato [21], but the appropriate irrigation level for late-season sweet potato remains unclear. Information on the responses of sweet potato to different irrigation levels is limited, as is information on optimum soil moisture during late-stage growth and storage root yield processes.

It is widely used to study the absorption and allocation of carbon by plants using ^13^C isotope tracer technique. [9,22,23]. Therefore, ^13^C leaf marker technology was used to quantitatively study the transfer and allocation of photosynthetic products in late-season sweet potato. The objectives of this study were to (1) explore the appropriate irrigation level in the late-season for high-yield, WUE, WP and economic return sweet potato production; and (2) explore the impact of the irrigation level on the source–sink of sweet potato, including the transfer and allocation of photosynthates thereto.

## 2. Results

### 2.1. Effects of Irrigation Levels on the Dynamics of Dry Matter Accumulation by Sweet Potato during the Late Season

Three samplings (S_1_–S_3_) were conducted during the two-year experiment (2021–2022). The results showed that irrigation treatments (T_1_–T_4_) significantly increased the above-ground and subsurface biomass of sweet potato compared with the non-irrigation treatment (T_0_) (*p* < 0.05). Above-ground biomass increased with increasing irrigation amount and was significantly higher in T_4_ than in the other treatments. However, with the increase in irrigation amount, the below-ground biomass showed a trend of initially increasing and then decreasing. When the irrigation amount exceeded that in T_2_, underground biomass began to decline although it was always higher than in T_0_. Over time, the root–shoot ratio of each treatment steadily increased, without a significant difference from T_0_ to T_2_; however, it was consistently higher in those treatments than in T_3_ and T_4_ (*p* < 0.05) (Table 1). The results showed that late-season irrigation increased both the above- and below-ground biomass of sweet potato, and that the irrigation level of T_2_ led to the accumulation of more below-ground biomass.

### 2.2. Effects of Irrigation Levels on Chlorophyll Content and Photosynthetic Parameters during Late-Season Sweet Potato Growth

**Figure 1:**
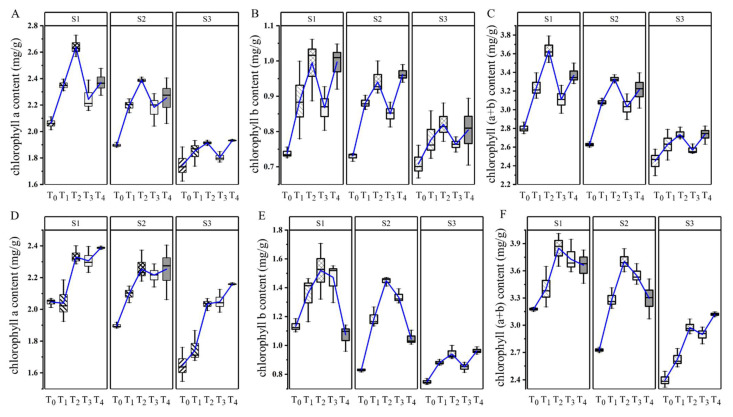
Dynamic effects of irrigation levels (T_0_–T_4_) on chlorophyll a content (**A**,**D**), chlorophyll b content (**B**,**E**) and total chlorophyll (a + b) (**C**,**F**) during late-season sweet potato growth in 2021 (**A**–**C**) and 2022 (**D**–**F**). S1–S3 represent 124, 134, and 144 days after transplantation, respectively.

Dynamic monitoring of the chlorophyll content of the functional leaves of sweet potato showed decreases in the contents of Chl-a, Chl-b and Chl-n over time, albeit to a lesser extent in the irrigated (T_1_–T_4_) than non-irrigated (T_0_) treatments. At all three samplings (S_1_–S_3_), irrigation (T_1_–T_4_) increased the contents of Chl-a, Chl-b and Chl-n in functional leaves over that measured at T_0_ during S_1_ and S_2_. The Chl-a content in the irrigation treatments increased in the order T_2_ > T_4_ > T_3_ > T_1_, whereas Chl-b and Chl-n were higher in T_2_ than in the other treatments. At S_3_, the contents of Chl-a, Chl-b and Chl-n decreased rapidly under the different treatments (Figure 1). Compared with non-irrigation (T_0_), late-season irrigation slowed the decline in chlorophyll content in functional leaves mainly at S_1_ and S_2_.

**Figure 2:**
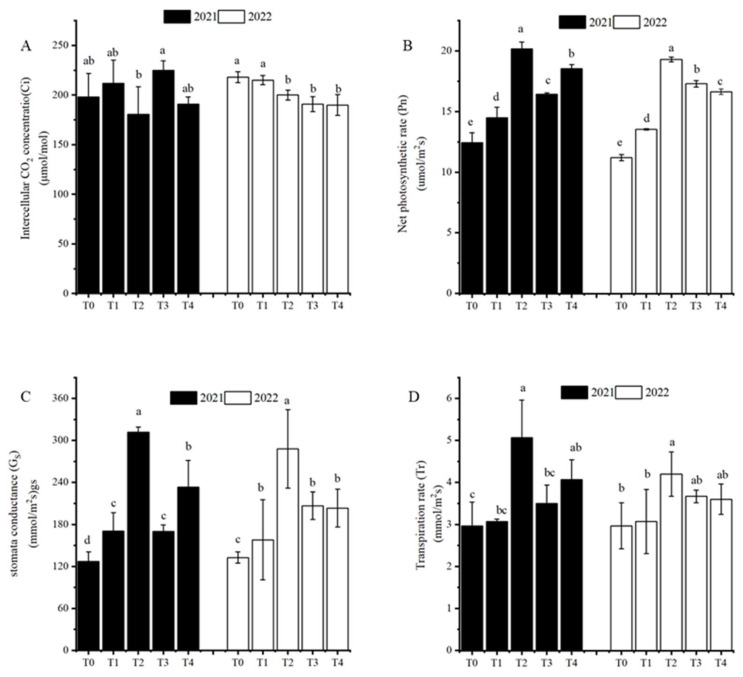
Effects of irrigation levels (T_0_–T_4_) on the photosynthetic parameters of sweet potato during its late-season growth. Intercellular carbon dioxide concentration (**A**), net photosynthetic rate (**B**), transpiration rate (**C**) and stomatal conductance (**D**). Different lowercase letters in the same period indicate a significant difference (*p* < 0.05); the same letter indicates no significant difference (*p* < 0.05).

Late-season irrigation had differential effects on the intercellular carbon dioxide concentration (Ci), net photosynthetic rate (Pn), transpiration rate (Tr) and stomatal conductance (Gs). In 2021 and 2022, Ci under T0 was significantly higher than under T_2_, but not significantly different from that in T_1_. All irrigation treatments (T_1_–T_4_) significantly increased the Pn and Gs of functional leaves compared with the non-irrigation treatment (T_0_) (*p* < 0.05). Pn and Gs were significantly higher under T_2_ than under T_1_, T_3_ and T_4_ (*p* < 0.05). Over the two-year period, Tr showed an increasing trend as the level of irrigation increased, but when the irrigation amount exceeded that of T_2_, the trend reversed and Tr decreased. Thus, late-season irrigation, especially at the T_2_ level, improved the net photosynthetic rate, stomatal conductance, and transpiration rate of functional leaves (Figure 2).

### 2.3. Effects of Irrigation Levels on ^13^C Accumulation and Allocation in Late-Season Sweet Potato Growth

**Figure 3:**
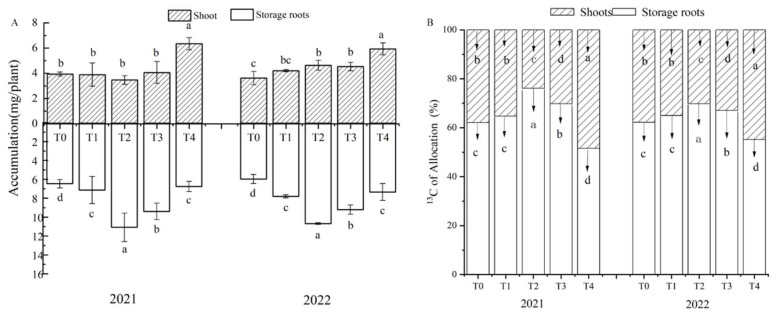
Effects of irrigation levels (T_0_–T_4_) on ^13^C accumulation (**A**) and the ^13^C allocation rate (**B**) in the shoots and storage roots of sweet potato in 2021 and 2022. Different lowercase letters in the same period indicate a significant difference (*p* < 0.05); the same letter indicates no significant difference (*p* < 0.05).

**Figure 4:**
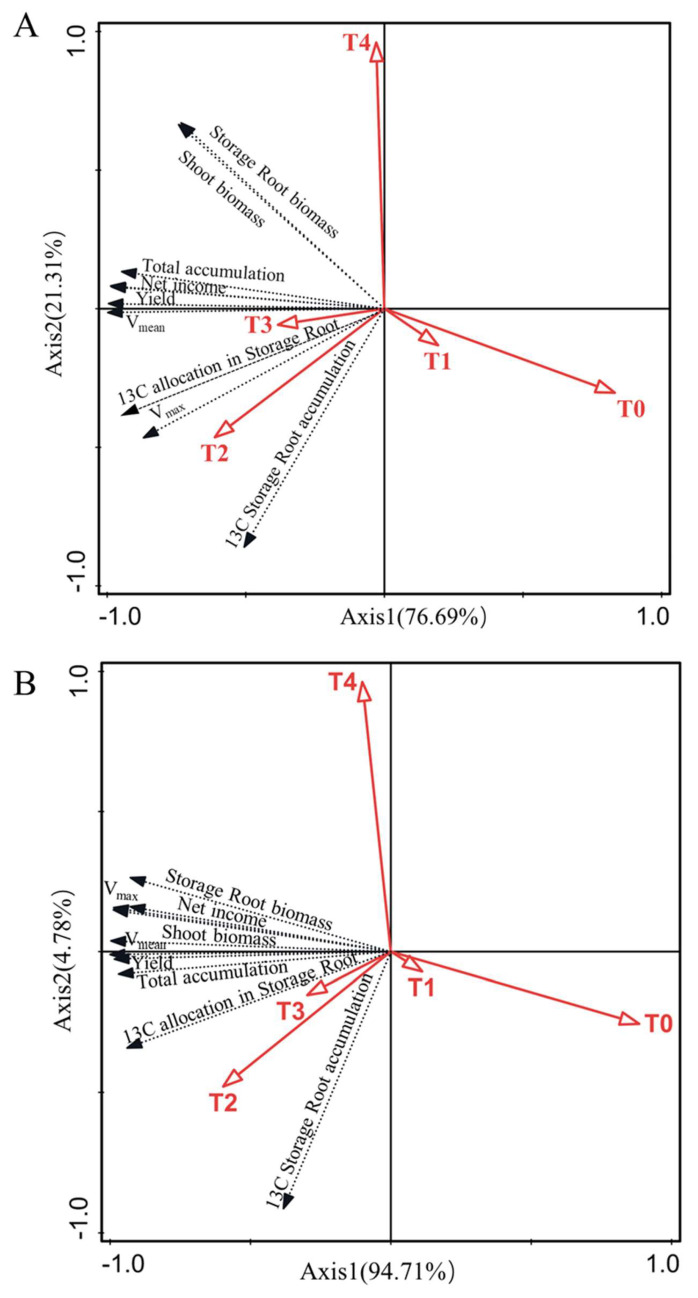
Redundancy analysis of irrigation level, yield, net profit, ^13^C accumulation and the ^13^C allocation rate in sweet potato shoots and storage roots based on a logistics function. (**A**) Data for the year 2021: axis 1, 76.69%; axis 2, 21.31%. (**B**) Data for the year 2022: axis 1, 94.71%; axis 2, 4.78%.

Supplemental irrigation at the bulking stage (124 days) significantly changed the accumulation and allocation rate of ^13^C in the shoots and storage roots of sweet potato (*p* < 0.05; Figure 3). ^13^C accumulation in sweet potato storage root was significantly higher under the different irrigation levels than under non-irrigation (T_0_) (*p* < 0.05). ^13^C accumulated in sweet potato storage root under irrigation in the order T_2_ > T_3_ > T_1_ > T_4_, and there was a significant difference between the T_2_ treatment and the other three treatments (*p* < 0.05). ^13^C accumulation in whole plants was similar to that in storage roots under the different treatments. By contrast, ^13^C accumulation in the above-ground parts of the plant did not significantly differ between the non-irrigation and irrigation treatments in 2021 (except compared to T_4_) (Figure 3A). Thus, ^13^C accumulation was higher in the irrigation than non-irrigation treatments in 2021 than 2022, and the difference was significant in the T_2_–T_4_ treatments. Under T_0_ and T_4_, the ^13^C allocation rate was significantly reduced in stems and leaves, but significantly increased in storage roots (Figure 3B). These results indicated that appropriate amounts of irrigation, exemplified by the T_2_ treatment, promote the allocation and accumulation of photosynthetic products in storage roots. Neither non-irrigation nor excessive irrigation was conducive to the allocation of ^13^C to storage roots. Redundancy analysis (RDA) showed that an irrigation level based on 75% evapotranspiration (T_2_) correlated well with total ^13^C accumulation by sweet potato, ^13^C accumulation by each organ, and the ^13^C allocation rate of the storage root (Figure 4).

### 2.4. Effects of Irrigation Level on the Dynamics of Dry Matter Accumulation in Storage Root

Dry matter accumulation by storage root was well-fitted using a logistics function (R^2^ > 0.99) (Table 2). The results showed that late-season irrigation changed the termination time and duration of rapid dry matter accumulation in sweet potato roots. In 2021, compared with T_0_ (non-irrigation), the end time (t_2_) of the rapid accumulation of storage root dry matter under irrigation was delayed by 3–4 days and the duration (T) was extended by 2–5 days. In 2022, the corresponding values were 3–6 days and 1 day. Moreover, late-season irrigation also affected the accumulation rate of dry matter. Compared with T_0_, the V_mean_ under irrigation (T_1_–T_4_) was significantly increased by 12.90–27.10% and 8.94–16.67% in 2021 and 2022, respectively (T_2_ > T_3_ > T_4_ > T_1_); however, the V_mean_ began to decline when the irrigation amount exceeded that of T_2_. The maximum accumulation rate (V_max_) in T_0_ was lower than that in T_1_–T_4_ (T_2_ > T_3_ > T_1_ > T_4_). The V_max_ in T_0_ was 13.69% and 17.56% lower than that in T_2_ in 2021 and 2022, respectively. The variation in the trend of the theoretical maximum accumulation (W_max_) was similar to that in the V_mean_, with irrigation (T_1_–T_4_) increasing the W_max_ compared with non-irrigation (T_0_). These results demonstrated the importance of irrigating sweet potato during the late-season of its growth. Irrigation maintained at the T_2_ and T_3_ levels prolonged the rapid accumulation time and increased the accumulation rate of dry matter, thus aiding achievement of the maximum theoretical yield (Table 2). The RDA showed that the V_mean_ and V_max_ were significantly positively correlated with the T_2_ and T_3_ treatments, but significantly negatively correlated with T_0_ and T_1_ (Figure 4).

### 2.5. Effects of Irrigation Level on the Yield and Water Use Efficiency of Sweet Potato

A two-way analysis of variance showed that irrigation and year had no significant interaction with yield, but irrigation and year had significant effects on yield. Compared with non-irrigation, the yield of sweet potato under the irrigated treatments was significantly increased by 13.30–28% and 11.7–20.0% in 2021 and 2022, respectively (*p* < 0.05). Among the irrigation treatments, the yield obtained with T_2_ was significantly higher than that obtained with T_4_ and T_1_, but did not significantly differ from that obtained with T_3_. The RDA showed significant positive correlations of production with the T_2_ and T_3_ treatments, while the correlations of production with the T_0_, T_1_ and T_4_ treatments were weak (Figure 4). Late-season irrigation increased the moisture content of the 0–60-cm soil layer, as well as the actual evapotranspiration of sweet potato. In the two-way analysis of variance, the changes in WUE and WP were significantly affected by irrigation, but were independent of year. WUE in the T_0_ and T_4_ treatments was significantly lower than in the T_1_–T_3_ treatments. Compared with the T_2_ treatment, the WUE of T_0_ and T_4_ decreased by 15.13% and 13.42%, respectively. WP was significantly higher under irrigation than non-irrigation (*p* < 0.05) conditions and was, on average, 19.13% higher in the T_2_ than T_0_ treatment. These results showed that moderate irrigation (T_2_) during the late-season growth can significantly increase the yield and water utilisation of sweet potato (Table 3).

### 2.6. Effects of Late-Season Irrigation on the Economic Benefits of Sweet Potato

Compared with T_0_ (non-irrigation), irrigation (T_1_−T_4_) improved the net profitability of sweet potato by $1377.74–2483.18 US ha^−1^ in 2021 and by $1764.00–3644.41 US ha^−1^ in 2022. Net profitability according to the level of irrigation was in the order T_2_ > T_3_ > T_4_ > T_1_. However, irrigation also increased the total investment, mainly due to the added costs of utilities and labour. Irrigation according to T_2_ increased the total yield (>150 g) by an average of 36.1% compared with T_0_ over the two years of the study. These results show that late-season irrigation can increase the commodity yield, and thus, net profitability of sweet potato (Table 4). According to the RDA, net profit correlated positively with irrigation, and there was a significant positive correlation between net profit and the T_2_ treatment (Figure 4).

## 3. Discussion

### 3.1. Effects of Late-Season Irrigation on Biomass, Chlorophyll Concentrations and Photosynthetic Parameters of Sweet Potato

In this study, field experiments were conducted to explore the effects of different irrigation levels on sweet potato during its late growth stage, and thus on its yield. Although the metabolic centre of sweet potato begins to shift from stem to storage root during the bulk expansion stage, insufficient or excessive leaf biomass accumulation will negatively affect the storage root yield of sweet potato [24]. In this study, dynamic sampling (S_1_–S_3_) of late-season sweet potato showed that irrigation significantly increased the plant’s above-ground and subsurface biomass over that achieved with no irrigation (Table 1). However, with increasing irrigation, the above-ground parts of sweet potato increased consistently, whereas below-ground biomass first increased and then decreased. This result suggested that an appropriate irrigation level can positively influence both above- and below-ground sweet potato parts, and therefore the yield.

Photosynthesis is the physiological basis of plant growth, and the absorption, transfer and transformation of light energy by plants are mainly completed through leaf chlorophyll [25]. However, plant leaves are extremely sensitive to the soil water conditions: an insufficient water supply will accelerate leaf senescence, resulting in a significant decline in the photosynthetic rate [26]. This study showed that late irrigation, especially at T_2_ irrigation level, could significantly slow the decline in chlorophyll content in functional leaves (Figure 1). This phenomenon may occur because irrigation promotes metabolic activity of chloroplasts, reduces the accumulation of reactive oxygen species, protects chloroplast membrane structure, and thus, reduces chlorophyll degradation [5,27]. This indicated that moderate irrigation of late-season sweet potato could slow down senescence of leaves and prolong the functional period of leaves [28,29,30]. In this study, the trends of Pn and Gs were similar, and under irrigation, both were significantly higher than in the non-irrigation treatment (Figure 2), in accordance with the previous findings of Ma, Morrison and Voldeng (1995) and Riekert and Robert (2008) that irrigation improves stomatal conductance, and thus, the photosynthetic rate [5,31]. This may reflect the increase in soil water content and leaf water potential, and stomatal openness (and thus, the carbon dioxide fixation rate induced by irrigation) [32], but may also be related to the degree of leaf senility. Haimeirong and Kubota (2003) and Niari and Abdollah (2012) found that stomatal conductance correlated negatively with the degree of leaf senility in sweet potato and wheat, respectively, and that irrigation slowed leaf senility; consequently, gas exchange in leaves was promoted and the photosynthetic rate increased [33,34]. The results showed that moderate irrigation of late-season sweet potato can prolong the functional period of the source and improve its photosynthetic efficiency. This is necessary to stimulate sink activity.

### 3.2. Effects of Late-Season Irrigation on the Relationships among ^13^C Allocation, Storage Root Enlargement and the Source–Sink Relationship of Sweet Potato

The photosynthate capacity of source leaves and transport capacity of photosynthate products from source to reservoir are important for storage root yield [35]. This study found that moderate irrigation significantly increased the total accumulation of ^13^C and the allocation rate in the storage roots of sweet potato as compared with non-irrigation (T_0_) and overirrigation (T_4_) (Figure 3), which was consistent with the rule also found in Li et al. (2021) for irrigation of mid-season sweet potato [9]. The results showed moderate irrigation improves the source–sink relationship, and thus, the transport of assimilates to storage. Non-irrigated (T_0_) and overirrigation (T_4_) were not conducive to the transfer and allocation of photosynthetic products to storage roots. This may be due to the fact that irrigation enhanced the photosynthate ability of the source and increased the fixation of photosynthate in the shoot [36]. No irrigation inhibits the growth of the source, leading to insufficient photosynthetic products to transport to storage roots. Over-irrigation leads to overgrowth of shoot and leaf, more allocation of photosynthetic products to metabolic sink (immature leaf), and less allocation to storage sink (root tuber) [37].

Source–sink coordination to achieve a balanced condition is a prerequisite for achieving a high crop yield [38]. Increasing sink strength is an important strategy to improve crop production potential, and sink strength can be expressed by the product of sink size and sink activity [35]. Ferreirah (2019) identified an S-shaped growth curve of dry matter accumulation in sweet potato storage roots [39]. A logistic function is a typical S-shaped growth curve that accurately quantifies crop growth [40,41]. Our fitting of the logistic function describing dry matter accumulation in storage root showed that, compared with non-irrigation, both a delay in the termination time of rapid dry matter accumulation in storage roots and prolongation of the process were achieved with irrigation. Late-season irrigation, especially at T_2_ irrigation level, significantly increased the rate of rapid dry matter accumulation (V_max_ and V_mean_) (sink activity) in storage roots (Table 2). The results showed that the transfer and allocation of photosynthate from source to sink under irrigation at late-season was that source increased the allocation of photosynthate to sink by stimulated activity of sink. The probable reason was that irrigation treatment prolonged functional period of source leaves as well as the net photosynthetic rate, thus increased the source strength [42].

### 3.3. Effects of Late-Season Irrigation on Sweet Potato Yield and Water Use Efficiency

As sweet potato is a moderately drought-tolerant crop, water management in the middle and late stages of its growth has been largely ignored. Consequently, the potential production capacity of sweet potato is often not fully developed and the yields are unreliable [43]. However, when the water supply is insufficient, sweet potato production is reduced due to drought stress, whereas when the water supply exceeds demand, water resources are wasted [43]. Determining the appropriate irrigation level for sweet potato will improve both its yield and WUE [44]. Studies using evapotranspiration to guide the irrigation of other crops have reported that an ET_c_ of 70–100% results in higher yields and better WUE. Shammout et al. (2018) obtained the highest yield of sweet pepper (*Capsicum annuum* L.) under 80% ET_c_ irrigation [45]. The field study of Yang (2017) showed that the yield and quality of potato, as well as WUE, were highest during the whole growth period when irrigation was based on 80% ET_c_ [46]. In a field study conducted in an arid area in northwest China, Zheng et al. (2012) found that 100% ET_c_ irrigation in the early and middle growth stages, and 40% ET_c_ irrigation in the late growth stage, increased the yield and WUE of onion [16]. In the study of Mattar et al. (2020), 100% ET_c_ irrigation improved tomato yield, fruit quality and irrigated WUE compared with 70% ET_c_ irrigation [17]. Our results showed that late-season irrigation increased sweet potato yield, WUE and WP, but when the irrigation amount exceeded 75% ET_c_ (T_2_), they declined. This is similar to the results obtained by Smittle et al. (1992) through field experiments and Gajanayake and Reddy (2016) through greenhouse experiments, which achieved the maximum yield under ET_c_ 76% and ET_c_ 72% irrigation, respectively [18,19]. Inconsistencies among studies may be due to differences in the type and variety of crops studied, as well in the environmental conditions, such as soil type and air temperature [47,48]. In summary, moderate irrigation (75% ET_c_) in the late-season not only improves irrigation WUE, but also results in high yields and should thus be implemented in agricultural production.

## 4. Materials and Methods

### 4.1. Experimental Site Description

**Figure 5:**
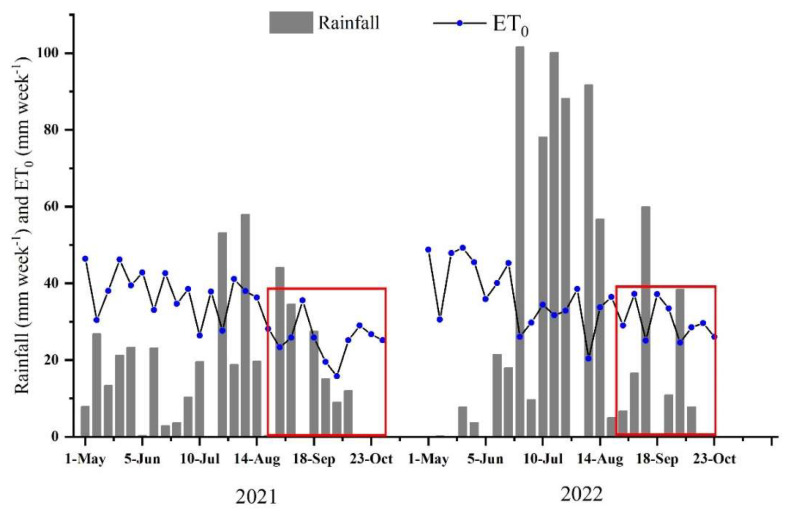
Precipitation and reference evapotranspiration during the whole sweet potato growth period, as determined in the study area from 2021 to 2022. The two red boxes represent rainfall and reference evapotranspiration during the 2021 and 2022 trials, respectively.

The field experiment was performed at a semi-humid site, i.e., the Nan village experimental base in Pingdu city, Shandong, China (36°53′ N, 120°13′ E) during 2021 and 2022. The climate is temperate semi-humid with an average annual precipitation of 630 mm, approximately 51% occurring between July and August. The local mean annual air temperature is 12.9 °C, according to the local average levels over the last 30 years (hppts://data.cma.cn (accessed on 10 August 2021)). A weather station was installed near the experimental area to monitor and record meteorological data. The mean air temperature was 22.47 °C and 22.37 °C, and the total net radiation was 2657.97 MJ m^−2^ and 2580.56 MJ m^−2^ in the 2021 and 2022 growing seasons, respectively. Precipitation and reference evapotranspiration during the 2021 and 2022 growing seasons are shown in Figure 5. The average sand, silt and clay contents in the 0−60-cm soil profile were measured with a laser particle size analyzer (BT-9300ST, Dandong baite, Liaoning, China) The soil consists of 19.5% clay (<0.002 mm), 43.2% silt (0.002–0.05 mm), and 37.3% sand (0.05–2.0 mm). It has a field water holding capacity of 28.5% and a permanent wilting coefficient of 12.1%. The average bulk density in the 0–60 cm soil layer is 1.3 g cm^−3^. The top layer of soil (20 cm) has a pH of 6.7, organic carbon of 11.7 g kg^−1^, total nitrogen of 60.4 g kg^−1^, available *p* of 19.4 mg kg^−1^, and available K of 66.5 mg kg^−1^, respectively.

### 4.2. Experimental Design and Treatments

The experimental variety of sweet potato was Yan 25, which is the main fresh food variety in northern China. The 2021 crop was planted on 20 May and the 2022 crop on 25 May. The plant is cultivated in raised bed, mulched film mode. In the late-season, five experimental treatments based on the evapotranspiration rate (ET_c_) were established: non-irrigation (T_0_), 33% ET_c_ (T_1_), 75% ET_c_ (T_2_), 100% ET_c_ (T_3_), and 125% ET_c_ (T_4_) (Table 5). A random block design was adopted with triplicates of each treatment. The area of each plot was 50 m^2^ (length: 10 m, width: 5 m) and the plot planting density was 200 plants (40,000 plants per hectare). It was used to dynamically monitor the volumetric water content of the soil layer between 0 and 60 cm. A soil moisture sensor (QY-800S, Qingyi, Hebei, China) was installed in each plot. The soil moisture sensor adopts a layered monitoring structure, and the underground soil is equipped with a soil moisture measuring point every 20 cm to dynamically monitor the soil moisture in the corresponding range. The applied fertilizer dose was 75 kg hm^−2^ for superphosphate, 150 kg hm^−2^ for urea, and 150 kg hm^−2^ for potassium sulphate.

### 4.3. Irrigation Treatments

The crop reference evapotranspiration rate (ET_0_) was calculated in the test area based on daily meteorological data, using the FAO 56 Penman–Monteith equation [49], adapted in Equation (1).

(1) $${\mathrm{ET}}_{0}=\frac{0.408\Delta \left(R_{n}-G\right)+\gamma \frac{900}{T+273}u_{2}{(e}_{x}{-e}_{a})}{\Delta +\gamma (1+0{.34u}_{2})}\;$$

where ET_0_ is the reference evapotranspiration rate (mm d^−1^); R_n_ is the net radiation (MJ m^−2^ day^−1^); G is the soil heat flux (MJ m^−2^ day^−1^); γ is the psychrometric constant (kPa °C^−1^); T is the air temperature at 2 m height (°C); u_2_ is the wind speed at 2 m height (m s^−1^); e_s_ is the saturation vapor pressure (kPa); e_a_ is the actual vapor pressure (kPa); and Δ is the slope of the vapor pressure curve (kPa °C^−1^). The crop growth coefficient K_c_ was calculated according to Equation (2), and the crop evapotranspiration rate (ET_c_) was obtained with Equation (3). When the soil water content in the 0–20 cm layer was less than 60% of the field capacity, irrigation was carried out after calculation of the irrigation amount according to Equations (4) and (5). Thereafter, supplementary irrigation was conducted every 7–15 days.

(2) $$K_{c}{=K}_{c}^{a}+\left[\left[0.04\left(u_{2}-2\right)-0.04\left({\mathrm{RH}}_{\min}-45\right)\right]\;\times \;{\left(\frac{h}{3}\right)}^{0.3}\right]$$

(3) $${\mathrm{ET}}_{c}{=\mathrm{ET}}_{0}{\;\times \;K}_{c}$$

(4) $${\mathrm{ET}}_{m}=\sum_{0}^{n}{\mathrm{ET}}_{c}\;$$

(5) $${I=\mathrm{ET}}_{m}-P,\;({\mathrm{ET}}_{m}\;\leq \;P,\;I=0)$$

where K_c_ is the crop growth coefficient, which varies according to growth stage (for sweet potato, values range from 0.65 to 1.15 [49]; $K_{c}^{a}$ is the crop coefficient calculated under standard conditions of the different growth stages (obtained from FAO 56 data); RH_min_ is the minimum daily relative mean humidity during a given growth stage (%); h is the average height of the crop during a given growth stage (m) and u_2_ is the wind speed at 2 m height (m s^−1^). In Equation (3), ET_c_ is the daily crop evapotranspiration (mm d^−1^). In Equation (4), ET_m_ is the cumulative evapotranspiration over the monitoring period and n is the number of days between irrigations. In Equation (5), I is the irrigation amount (mm) and P is the effective rainfall (mm); when the rainfall is >5 mm within 24 h, it is the effective rainfall.

### 4.4. Data Collection

#### 4.4.1. Determination of Sweet Potato Biomass

We collected samples on experimental days 36, 100, 115, 124, 134, 144, 150 (harvest date 2022), and 160 (harvest date 2021). We randomly sampled 6 whole sweet potatoes under each treatment. Shoots and storage roots were separated and taken to the laboratory for determination of fresh weights and partially decomposed; they were dried at 80 °C for 48 h to constant weight.

#### 4.4.2. Determination of Chlorophyll Content

Samples were obtained on experimental days 124, 134 and 144. Six whole sweet potatoes were randomly sampled under each treatment and processed using the acetone ethanol solution colorimetric method. Briefly, a portion (0.1 g) of the fourth functional leaf was added to 50 mL of acetone ethanol solution (4.5:4.5:1) in the dark for 48 h, after which absorbance was determined at wavelengths of 663 and 645 nm. The contents of chlorophyll a (Chl-a), chlorophyll b (Chl-b) and total chlorophyll (Chl-n) were calculated according to Equations (6)–(8), respectively:

(6) $$C_{a}=12{.72A}_{663}-2{.69A}_{645}$$

(7) $$C_{b}=22{.88A}_{645}-4{.67A}_{663}$$

(8) $$C_{T}{=C}_{a}{+C}_{b}=20{.19A}_{645}-8{.05A}_{663}$$

where C_a_ is the Chl-a content (mg g^−1^), C_b_ is the Chl-b content (mg g^−1^), C_T_ is the Chl-n content (mg g^−1^), and A_663_ and A_645_ are the absorbances of the extracted chloroplast pigment at 663 and 645 nm, respectively.

#### 4.4.3. Determination of Photosynthetic Parameters of Sweet Potato Leaves

Seven days after the first supplemental irrigation, six plants were randomly selected from each treatment. The net photosynthetic rate (Pn), stomatal conductance (Gs), transpiration rate (Tr), and the leaf internal CO_2_ concentration (Ci) of the fourth and fifth functional leaves of each plant were measured by CIRAS-3 portable photosynthesis system (CIRAS-3, Hansha, Boston, MA, USA).

#### 4.4.4. Logistic Regression Equation and Parameters

Samples were obtained on experimental days 36, 100, 115, 144, and 150, with six whole sweet potatoes randomly sampled under each treatment. Dry matter accumulation in the storage root of sweet potato increased with time and could be described by a typical S-shaped growth curve. A logistic function was used to fit the dry matter accumulation dynamics of storage root (Equation (9)).

(9) $$W=\frac{W_{\max}}{\left({1+\mathrm{ae}}^{-\mathrm{bt}}\right)}$$

where t is the sampling day (d), W is the dry matter of sweet potato tuber (g plant^−1^), e is a mathematical constant (e = 2.71828), W_max_ is the maximum dry matter of sweet potato tuber (g plant^−1^) and parameters *a* and *b* are related to the environmental conditions in the field. The values of a and b, which were derived from the logistic regression, can be used as shown in Equations (11)–(15) to calculate the initiation (t_1_) and termination (t_2_) times of dry matter accumulation by sweet potato tuber, the duration of these periods (T), and the maximum rate (V_max_) and average rate (V_mean_) of rapid dry matter accumulation by sweet potato tuber:

(10) $$t_{1}=-\frac{1}{b}\ln\frac{2+\sqrt{3}}{a}$$

(11) $$t_{2}=-\frac{1}{b}\ln\frac{2-\sqrt{3}}{a}$$

(12) $$V_{\max}=\frac{W_{\max}\times b}{4}$$

(13) $$V_{\mathrm{mean}}=\frac{W_{\max}\times b}{\ln9a}$$

(14) $${T=t}_{1}{+t}_{2}$$

#### 4.4.5. ^13^C labelling of Sweet Potato Leaves and Determination of the ^13^C Accumulation and Allocation Ratio

Seven days after the first supplemental irrigation of sweet potato (122 days), the plants were labelled with ^13^CO_2_ (^13^C 99%). Three plants with uniform growth were selected from each plot, and the fourth and fifth leaves of the longest stem were chosen for labelling. The leaves to be marked were sealed in a PVC transparent plastic film bag with a volume of ~40 mL, into which 5 mL of ^13^CO_2_ gas was injected from a syringe. Photosynthesis was allowed to proceed for 2 h, after which the transparent plastic bags were removed; the whole plant was harvested 2 days later. The harvested underground parts and storage roots were dried and crushed. Isotope ratio mass spectrometry was used to determine δ^13^C and total carbon content (C%). The latter measurements were carried out at the stable isotope laboratory of the Department of Plant Science of the University of California (Davis, CA, USA). The calculations are shown in Equations (15)–(18).

(15) $$\mathrm{Fi}\left(\%\right)=\frac{\left(\delta^{13}C+1000\right){\times R}_{\mathrm{Standard}}}{\left[\left(\delta^{13}C+1000\right){\times R}_{\mathrm{Standard}}+1000\right]}\times 100\%$$

(R_Standard_ is the carbon isotope standard ratio, R_Standard_ = 0.0112372)

(16) C13i(μg/g)=Total CSample Weight×Fi−FNattural×104

total carbon accumulation (g) = biomass(g) × total carbon content of sweet potato organs (%)(17)

(18) C13 allocation rate%=C13 accumulation in the organtotalC13 accumlation in the plant×100%

#### 4.4.6. Sweet Potato Evapotranspiration and Water Use Efficiency

A soil moisture sensor (QY-800S, Qingyi, Hebei, China) was used to measure the soil moisture content at the 60-cm soil layer. Soil measurements were performed before planting, after harvest, and before and after irrigation. Total water consumption or actual evapotranspiration (ET_a_, mm) was calculated during the growing season using the soil water balance equation (Equation (19)):

(19) $${\mathrm{ET}}_{a}{=I+P+C}_{R}{-R}_{F}{-D}_{P}\pm \Delta S$$

where ET_a_ is the actual evapotranspiration (mm) during the growing season, I is the amount of irrigation water applied (mm), P is the precipitation (mm), C_R_ is the capillary rise (mm), D_P_ is the percolation (mm), R_F_ is the runoff (mm) and ΔS is the change in soil moisture content (mm). C_R_ was considered to be zero because the groundwater table was 2 m below the surface; runoff was also assumed to be insignificant because the field was flat, and D_P_ was considered negligible because the soil water content below 60 cm did not reach field capacity on any sampling date.

Crop water use efficiency (WUE, kg ha^−1^ mm^−1^) was calculated as the ratio of the sweet potato yield per unit area to total water consumption for the whole season. Water productivity (WP, kg ha^−1^ mm^−1^) was calculated as the ratio of sweet potato yield per unit area to the total irrigation and precipitation amount for the whole season, as shown in Equations (20) and (21):

(20) $$\mathrm{WUE}=\frac{Y}{{\mathrm{ET}}_{a}}\;$$

(21) $$\mathrm{WP}=\frac{Y}{\mathrm{TWU}}\;$$

where Y is the sweet potato yield (kg ha^−1^), ET_a_ (mm) is the corresponding actual amount of crop evapotranspiration during each growing season, and TWU (mm) is the total water irrigation and precipitation amount.

#### 4.4.7. Economic Analysis

Economic return was estimated as shown in Equations (22)–(26):

Economic return ($US ha^−1^) = Storage roots yield benefit ($US ha^−1^) − Total cost ($US ha^−1^) (22)

Storage roots yield benefit ($US ha^−1^) = Storage roots yield (kg ha^−1^) × Sweet potato price (US $kg^−1^) (23)

Toal cost ($US ha^−1^) = land use fee ($US ha^−1^) + machinery operating cost ($US ha^−1^) + irrigation equipment cost ($US ha^−1^) + fertilizer cost ($US ha^−1^) + pesticide cost ($US ha^−1^) + seedling cost (US $ha^−1^) + labour cost ($US h^−1^) + electricity cost (m^3^ ha^−1^) + water cost (m^3^ ha^−1^) (24)

Electricity cost (m3 ha^−1^) = irrigation amount (m^3^) × electricity price ($US m^−3^) (25)

Water cost (m^3^ ha^−1^) = irrigation amount (m^3^) × water price ($US m^−3^) (26)

### 4.5. Data Analyses

In order to observe the differences between the treatments, SPSS 20.0 was used to analyze the variance of each indicator data to compare the effective difference between the treatments. The LSD method compared the significance of the difference between the averages. The logistic equation was performed with Curve Expert Professional 2.6.5 software. In order to clarify the correlation between soil moisture, chlorophyll content in leaves, photosynthetic parameters, characteristic parameters of logistics model, ^13^C accumulation, yield and water use efficiency, RDA (redundancy analysis) was performed using Canoco 5.0.

## 5. Conclusions

In northern China, timely irrigation of late-season sweet potato not only reduced the soil water deficit, but also enhanced the physiological functions of sweet potato. Late-season irrigation prolonged the functional period of functional leaves, promoted dry matter accumulation, increased the yield, and resulted in greater economic benefits. The 75% ET_c_ (T_2_) moderate irrigation effectively promoted the source–sink balance, enhanced the source photosynthetic rate and stimulated the sink activity, such that more photosynthate is allocated to the storage sink, resulting in a high yield. Over-irrigation at 125% ET_c_ (T_4_) increased the source capacity, but caused more photosynthates to be allocated to the metabolic sink supporting the growth of young leaves, which was not conducive to a good source–sink balance of sweet potato and did not increase production, causing waste of water resources. Thus, 75% ET_c_ (T_2_) irrigation should be implemented for late-season sweet potato, as it improves the yield of this important crop, and by extension, farmers’ incomes.

## Figures and Tables

**Table 1:** Effects of irrigation levels on above- and below-ground dry matter mass in the late-season stage of sweet potato growth.

Year	Treatment	Shoot Biomass (g/plant)			Storage Root Biomass (g/plant)			Storage Root/Shoot		
		S_1_	S_2_	S_3_	S_1_	S_2_	S_3_	S_1_	S_2_	S_3_
2021	T_0_	90.00 ± 2.53 d	90.67 ± 2.53 c	89.55 ± 1.97 d	174.12 ± 5.72 c	186.62 ± 4.95 d	200.74 ± 0.39 d	1.93 ± 0.12 ab	2.06 ± 0.05 a	2.24 ± 0.04 a
	T_1_	106.40 ± 3.15 c	109.68 ± 3.7 b	113.55 ± 5.28 c	189.58 ± 9.50 bc	211.82 ± 5.59 c	227.57 ± 0.94 c	1.78 ± 0.11 abc	1.93 ± 0.02 ab	2.00 ± 0.09 bc
	T_2_	113.20 ± 6.74 bc	119.68 ± 6.74 b	121.00 ± 4.63 bc	228.76 ± 5.76 a	239.27 ± 5.73 a	257.00 ± 0.78 a	2.02 ± 0.10 a	2.00 ± 0.10 a	2.12 ± 0.09 ab
	T_3_	123.07 ± 3.61 b	128.35 ± 5.20 b	130.11 ± 4.39 b	204.50 ± 3.83 b	227.34 ± 3.30 ab	244.35 ± 1.11 ab	1.66 ± 0.07 b	1.77 ± 0.07 b	1.88 ± 0.05 c
	T_4_	137.47 ± 5.15 a	141.05 ± 2.62 a	143.00 ± 3.24 a	208.44 ± 3.48 b	219.62 ± 4.47 bc	235.73 ± 0.80 bc	1.52 ± 0.08 c	1.56 ± 0.06 c	1.65 ± 0.04 d
2022	T_0_	100.66 ± 6.45 d	101.53 ± 4.39 c	103.86 ± 3.41 c	133.67 ± 9.92 b	143.67 ± 8.57 b	155.41 ± 3.17 c	1.34 ± 0.18 a	1.42 ± 0.07 a	1.50 ± 0.04 a
	T_1_	111.37 ± 4.63 cd	112.30 ± 5.54 c	114.13 ± 9.14 c	147.63 ± 16.45 a	159.27 ± 1.87 a	173.57 ± 2.45 b	1.24 ± 0.16 a	1.42 ± 0.06 a	1.53 ± 0.11 a
	T_2_	121.32 ± 11.78 bc	125.68 ± 10.45 b	128.82 ± 7.65 b	149.31 ± 10.13 a	162.15 ± 9.92 a	178.70 ± 2.71 a	1.16 ± 0.10 ab	1.29 ± 0.09 ab	1.39 ± 0.10 ab
	T_3_	128.56 ± 6.24 ab	130.53 ± 8.49 ab	134.27 ± 7.60 ab	149.23 ± 6.64 a	161.56 ± 5.71 a	176.68 ± 1.48 ab	1.08 ± 0.09 ab	1.24 ± 0.07 bc	1.32 ± 0.06 bc
	T_4_	140.80 ± 8.15 a	142.54 ± 4.08 a	145.20 ± 5.61 a	151.56 ± 6.03 a	161.77 ± 7.73 a	174.73 ± 0.51 ab	1.33 ± 0.11 b	1.14 ± 0.09 c	1.20 ± 0.05 c

Note: S_1_–S_3_ represent 124, 134, and 144 days after transplantation, respectively. Data are expressed as mean ± SE. Different lowercase letters in the same period indicate a significant difference (*p* < 0.05); the same letter indicates no significant difference (*p* < 0.05).

**Table 2:** Characteristic parameters of the dynamic curve of dry matter accumulation in sweet potato roots.

Year	Treatment (ET_c_)	Model	t_1_	t_2_	T	V_mean_	V_max_	W_max_
			d	d	d	g d^−1^	g d^−1^	g plant^−1^
2021	T_0_	Y = 205.93/(1 + 1431,594.12e^−0.1232^X) R^2^ = 0.9979	107	129	22	1.55	6.18	205.93
	T_1_	Y = 238.60/(1 + 625,644.62e^−0.1138^X) R^2^ = 0.9983	109	133	24	1.75	6.56	238.60
	T_2_	Y = 280.66/(1 + 218,869.20e^−0.1022^X) R^2^ = 0.9934	108	133	25	1.97	7.16	280.66
	T_3_	Y = 262.62/(1 + 290,040.10e^−0.1056^X) R^2^ = 0.9986	107	132	25	1.89	6.89	262.62
	T_4_	Y = 252.81/(1 + 131,844.28e^−0.0993^X) R^2^ = 0.9986	106	133	27	1.79	6.26	252.81
2022	T_0_	Y = 154.24/(1 + 342,7650.28e^−0.1374^X) R^2^ = 0.9941	100	119	19	1.23	5.30	154.13
	T_1_	Y = 173.87/(1 + 7,382,425.82e^−0.1393^X) R^2^ = 0.9930	104	123	19	1.34	6.06	171.87
	T_2_	Y = 182.62/(1 + 6,612,877.62e^−0.1399^X) R^2^ = 0.9940	103	122	19	1.43	6.39	177.62
	T_3_	Y = 177.75/(1 + 5,899,178.32e^−0.1384^X) R^2^ = 0.9962	103	122	19	1.38	6.15	175.75
	T_4_	Y = 175.37/(1 + 7,821,166.95e^−0.1380^X) R^2^ = 0.9970	105	125	20	1.34	6.05	175.37

Note: t_1_ and t_2_ are, respectively, the initiation and termination times of dry matter accumulation by storage root per plant (d); T is the duration of dry matter accumulation by storage root per plant (d); V_max_ is the maximum rate of dry matter accumulation by storage root per plant (g d^−1^); V_meam_ is the average rate of dry matter accumulation by storage root per plant (g d^−1^); and W_max_ is the maximum theoretical dry matter accumulation by storage root per plant (g plant^−1^).

**Table 3:** Seasonal components of water balance, including actual evapotranspiration (ETa), sweet potato yield, water use efficiency (WUE) and water productivity (WP), for all irrigation treatments in 2021 and 2022.

Year	Treatment	I (mm)	P (mm)	ΔS (mm)	Eta (mm)	Yield (t ha^−1^)	WUE (kg ha^−1^ mm^−1^)	WP (kg ha^−1^ mm^−1^)
2021	T_0_	13.50	418.48	24.02	456.00	40.14 ± 0.39 d	88.03 ± 0.84 c	92.93 ± 0.89 d
	T_1_	24.40	418.48	26.31	469.20	45.50 ± 0.94 c	96.97 ± 2.01 b	102.73 ± 2.13 b
	T_2_	38.30	418.48	32.69	489.50	51.38 ± 1.62 a	104.97 ± 1.59 a	112.48 ± 1.70 a
	T_3_	46.60	418.48	39.50	504.60	48.87 ± 1.11 ab	96.86 ± 2.20 b	105.08 ± 2.38 bc
	T_4_	54.90	418.48	54.65	528.00	46.77 ± 0.80 bc	88.57 ± 1.52 c	98.80 ± 1.69 c
2022	T_0_	13.50	465.36	25.94	504.80	38.86 ± 0.15 c	76.98 ± 1.79 d	81.15 ± 1.89 c
	T_1_	18.50	465.36	27.87	511.70	43.41 ± 0.61 b	84.83 ± 0.97 ab	89.72 ± 1.03 b
	T_2_	24.90	465.36	31.43	521.70	46.65 ± 0.91 a	89.42 ± 0.16 a	95.15 ± 0.17 a
	T_3_	28.70	465.36	39.32	533.40	44.15 ± 0.50 ab	82.77 ± 1.94 b	89.36 ± 2.10 b
	T_4_	32.60	465.36	45.50	543.50	43.63 ± 0.08 b	80.28 ± 0.28 c	87.62 ± 0.30 b
Significance								
Water(W)						**	*	*
Year(Y)						**	NS	NS
W × Y						NS	NS	NS

Data are expressed as mean ± SE. Different lowercase letters for the same period indicate a significant difference (*p* < 0.05); the same letter indicates no significant difference (*p* < 0.05). * and ** are significant at *p* < 0.05 and *p* < 0.01, respectively. NS is not significance.

**Table 4:** Effects of irrigation level on the economic benefits of sweet potato in late 2021 and 2022.

Year	Treatment	Input Value of Consumable Items ($US ha^−1^)						Total Input	Output Value of Consumable Items ($US ha^−1^)		Total Output	Output/Input	Net Income ($US ha^−1^)
		Land Use Fee and Seedling Cost	Machinery Operating Cost	Irrigation Equipment Cost	Pesticide and Fertilizer Cost	Electricity and Water Cost	Labour Cost	($US ha^−1^)	≥150 g	<150 g		($US ha^−1^)	
2021	T_0_	1470	546	210	546	10.40	697.54	3479.93	9562.77	10.58	9573.343	2.75	6093.41
	T_1_	1470	546	210	546	18.79	701.20	3491.99	10,953.7	9.44	10,963.14	3.14	7471.15
	T_2_	1470	546	210	546	29.49	705.87	3507.36	12,066.52	17.44	12,083.95	3.45	8576.59
	T_3_	1470	546	210	546	35.88	708.66	3516.54	11,728.80	10.95	11,739.75	3.34	8223.21
	T_4_	1470	546	210	546	42.27	711.45	3525.72	11,233.97	10.27	11,244.24	3.19	7718.52
2022	T_0_	1470	546	210	546	10.40	697.54	3479.93	7999.79	85.85	8085.643	2.32	4605.71
	T_1_	1470	546	210	546	14.25	699.22	3485.46	9773.05	82.13	9855.172	2.83	6369.71
	T_2_	1470	546	210	546	19.17	701.37	3492.54	11,673.70	68.97	11,742.66	3.36	8250.12
	T_3_	1470	546	210	546	22.10	702.64	3496.74	10,958.03	66.75	11,024.78	3.15	7528.04
	T_4_	1470	546	210	546	25.10	703.95	3501.06	10,858.63	65.48	10,924.11	3.12	7423.06

Note: fixed production inputs include the land use fee, $630 US ha^−1^; machinery operation, $546 US ha^−1^; irrigation equipment, $210 US ha^−1^; pesticide, $126 US ha^−1^; fertiliser, $420 US ha^−1^ and seedlings, $840 US ha^−1^. The price of sweet potato with a weight ≥150 g (marketable sweet potato) was $0.25 US kg^−1^ (2021) and $0.34 US kg^−1^ (2022). The price of sweet potato with a weight < 150 g was $0.056 US kg^−1^ (2021 and 2022). Labour cost, $2.24 US h^−1^; electricity, $0.035 US m^−3^; water, $0.042 US m^−3^. Data required for the economic return calculation were collected from a survey of local farms conducted during both study years.

**Table 5:** Dates and crop coefficient (K_c_) values for each sweet potato growth stage and the total amount of water applied during the study period.

Growth Stages	Duration (days)	$K_{c}^{a}$	(K_c_) Adjusted	Water Applied (mm)				
				T_0_	T_1_	T_2_	T_3_	T_4_
2021	20 May–27 October							
Planting	1			13.50	13.50	13.50	13.50	13.50
initial	41	0.50		81.50	81.50	81.50	81.50	81.50
Mid-season	51	1.15		165.70	165.70	165.70	165.70	165.70
Late season	67	0.65	0.60	172.20	181.18	196.10	204.37	212.64
Seasonal	160			431.98	442.90	456.80	465.07	473.34
2022	25 May–23 October							
Planting	1			13.50	13.50	13.50	13.50	13.50
initial	40	0.50		105.60	105.6	105.60	105.60	105.60
Mid-season	50	1.15		206.00	206.00	206.00	206.00	206.00
Late-season	59	0.65	0.63	153.76	158.79	165.19	169.00	172.81
Seasonal	150			478.86	483.89	490.29	494.10	497.91

## Data Availability

Not applicable.

## References

[1] Sonnewald U., Fernie A.R. (2018). Next-generation strategies for understanding and influencing source-sink relations in crop plants. Curr. Opin. Plant Biol..

[2] Gajanayake B., Reddy K.R., Shankle M.W., Arancibia R.A. (2014). Growth, developmental, and physiological responses of two sweetpotato (*Ipomoea batatas* L. [Lam]) cultivars to early season soil moisture deficit. Sci. Hortic..

[3] Reddy A.R., Chaitanya K.V., Vivekanandan M. (2004). Drought-induced responses of photosynthesis and antioxidant metabolism in higher plants. J. Plant Physiol..

[4] Zhao C.X., Guo L.Y., Jaleel C.A., Shao H.B., Yang H.B. (2008). Prospectives for applying molecular and genetic methodology to improve wheat cultivars in drought environments. C. R. Biol..

[5] Van Heerden P.D.R., Laurie R. (2008). Effects of prolonged restriction in water supply on photosynthesis, shoot development and storage root yield in sweet potato. Physiol. Plant..

[6] Gajanayake B., Reddy K.R., Shankle M.W., Arancibia R.A., Villordon A.O. (2014). Quantifying Storage Root Initiation, Growth, and Developmental Responses of Sweetpotato to Early Season Temperature. Agron. J..

[7] Kwak S.S. (2019). Biotechnology of the sweetpotato: Ensuring global food and nutrition security in the face of climate change. Plant Cell Rep..

[8] Villordon A., LaBonte D., Solis J., Firon N. (2012). Characterization of Lateral Root Development at the Onset of Storage Root Initiation in ‘Beauregard’ Sweetpotato Adventitious Roots. HortScience.

[9] Li S., Zhao L., Sun N., Liu Q., Li H. (2021). Photosynthesis product allocation and yield in sweetpotato with different irrigation levels at mid-season. Agric. Water Manag..

[10] Zhang H.Y., Xie B.T., Duan W.X., Dong S.X., Wang B.Q., Zhang L.M., Shi C.Y. (2018). Effects of drought stress at different growth stages on photosynthetic efficiency and water consumption characteristics in Sweet potato. Appl. Ecol..

[11] Rankine D.R., Cohen J.E., Taylor M.A., Coy A.D., Simpson L.A., Stephenson T., Lawrence J.L. (2015). Parameterizing the FAO AquaCrop Model for Rainfed and Irrigated Field-Grown Sweet Potato. Agron. J..

[12] Zierer W., Rüscher D., Sonnewald U., Sonnewald S. (2021). Tuber and Tuberous Root Development. Annu. Rev. Plant Biol..

[13] Lewthwaite S.L., Triggs C.M. (2012). Sweet potato cultivar response to prolonged drought. Proceedings of the 42nd Agronomy Society Conference.

[14] Ngigi S.N., Savenije H.H., Thome J.N., Rockström J., de Vries F.P. (2005). Agro-hydrological evaluation of on-farm rainwater storage systems for supplemental irrigation in Laikipia district, Kenya. Agric. Water Manag..

[15] Nagaz K., El Mokh F., Alva A.K., Masmoudi M.M., Ben Mechlia N. (2016). Potato Response to Different Irrigation Regimes Using Saline Water. Irrig. Drain..

[16] Zheng J., Huang G., Wang J., Huang Q., Pereira L.S., Xu X., Liu H. (2012). Effects of water deficits on growth, yield and water productivity of drip-irrigated onion (*Allium cepa* L.) in an arid region of Northwest China. Irrig. Sci..

[17] Mattar M.A., Zin El-Abedin T.K., Alazba A.A., Al-Ghobari H.M. (2020). Soil water status and growth of tomato with partial root-zone drying and deficit drip irrigation techniques. Irrig. Sci..

[18] Thompson P.G., Smittle D.A., Hall M.R. (1992). Relationship of Sweetpotato Yield and Quality to Amount of Irrigation. HortScience.

[19] Gajanayake B., Reddy K.R. (2016). Sweetpotato Responses to Mid- and Late-Season Soil Moisture Deficits. Crop Sci..

[20] Rao R., Li Y. (2003). Management of Flooding Effects on Growth of Vegetable and Selected Field Crops. HortTechnology.

[21] Siqinbatu Y.K., Hirai H., Endo R., Shibuya T. (2013). Effects of water contents and CO_2_ concentrations in soil on growth of sweet potato. Field Crop. Res..

[22] Huang H., Zhou M., Liang B., Xiang D., Li H. (2022). Effects of atmospheric CO_2_ on canopy uptake of gaseous ammonia by tomato (*Lycopersicum esculentum* Mill.) in polytunnel vegetable production systems. Sci. Hortic..

[23] Huan L., Jin-Qiang W., Qing L. (2020). Photosynthesis product allocation and yield in sweet potato with spraying exogenous hormones under drought stress. J. Plant Physiol..

[24] Pérez-Pazos J.V., Rosero A., Martínez R., Pérez J., Morelo J., Araujo H., Burbano-Erazo E. (2021). Influence of morpho-physiological traits on root yield in sweet potato (*Ipomoea batatas* Lam.) genotypes and its adaptation in a sub-humid environment. Sci. Hortic..

[25] Liu J., Friebe V.M., Frese R.N., Jones M.R. (2020). Polychromatic solar energy conversion in pigment-protein chimeras that unite the two kingdoms of (bacterio)chlorophyll-based photosynthesis. Nat. Commun..

[26] Saeedipour S., Moradi F. (2011). Effect of Drought at the Post-anthesis Stage on Remobilization of Carbon Reserves and Some Physiological Changes in the Flag Leaf of Two Wheat Cultivars Differing in Drought Resistance. J. Agric. Sci..

[27] Igamberdiev A.U., Mikkelsen T.N., Ambus P., Bauwe H., Lea P.J., Gardeström P. (2004). Photorespiration Contributes to Stomatal Regulation and Carbon Isotope Fractionation: A Study with Barley, Potato and Arabidopsis Plants Deficient in Glycine Decarboxylase. Photosynth. Res..

[28] Verma V., Foulkes M.J., Worland A.J., Sylvester-Bradley R., Caligari P.D.S., Snape J.W. (2004). Mapping quantitative trait loci for flag leaf senescence as a yield determinant in winter wheat under optimal and drought-stressed environments. Euphytica.

[29] Xu X., Zhang Y., Li J., Zhang M., Zhou X., Zhou S., Wang Z. (2018). Optimizing single irrigation scheme to improve water use efficiency by manipulating winter wheat sink-source relationships in Northern China Plain. PLoS ONE.

[30] Gouveia C.S., Ganança J.F., Slaski J., Lebot V., de Carvalho M.Â.P. (2019). Variation of carbon and isotope natural abundances (delta(15)N and delta(13)C) of whole-plant sweet potato (*Ipomoea batatas* L.) subjected to prolonged water stress. J. Plant Physiol..

[31] Ma B.L., Morrison M.J., Voldeng H.D. (1995). Leaf Greenness and Photosynthetic Rates in Soybean. Crop Sci..

[32] Haimeirong F., Kubota F., Yoshimura Y. (2002). Estimation of Photosynthetic Activity from the Electron Transport Rate of Photosystem 2 in a Film-Sealed Leaf of Sweet Potato, *Ipomoea batatas* Lam. Photosynthetica.

[33] Haimeirong F., Kubota F. (2003). The Effects of Drought Stress and Leaf Ageing on Leaf Photosynthesis and Electron Transport in Photosystem 2 in Sweet Potato (Ipomoea batatas Lam.) Cultivars. J. Photosynth..

[34] Khamssi N.N., Najaphy A. (2012). Comparison of photosynthetic components of wheat genotypes under rain-fed and irrigated conditions. Photochem. Photobiol..

[35] White A.C., Rogers A., Rees M., Osborne C.P. (2016). How can we make plants grow faster? A source–sink perspective on growth rate. J. Exp. Bot..

[36] Laurie R.N., Laurie S.M., Du Plooy C.P., Finnie J.F., Van Staden J. (2015). Yield of Drought-Stressed Sweet Potato in Relation to Canopy Cover, Stem Length and Stomatal Conductance. J. Agric. Sci..

[37] Ning Y., Ma H., Zhang H., Wang J., Xu X., Zhang Y. (2015). Response of Sweetpotato in Source-Sink Relationship Establishment, Expanding, and Balance to Nitrogen Application Rates. Acta Agron. Sin..

[38] Smith A.M., Stitt M. (2007). Coordination of carbon supply and plant growth. Plant Cell Environ..

[39] Ferreira M.A.M., Andrade V.C., Oliveira A.J., Ferreira E.A., Brito O.G., Silva L.R. (2019). Physiological characterization of plant growth in sweet potato. Hortic. Bras..

[40] Xue J., Zhao Y., Gou L., Shi Z., Yao M., Zhang W. (2016). How High Plant Density of Maize Affects Basal Internode Development and Strength Formation. Crop Sci..

[41] Xiangbei D., Lingcong K., Min X., Xinyue Z. (2019). Split application improving sweetpotato yield by enhancing photosynthetic and sink capacity under reduced nitrogen condition. Field Crop. Res..

[42] Keutgen N., Mukminah F., Roeb G.W. (2002). Sink strength and photosynthetic capacity influence tuber development in sweet potato. J. Hortic. Sci. Biotechnol..

[43] Daryanto S., Wang L., Jacinthe P.A. (2016). Drought effects on root and tuber production: A meta-analysis. Agric. Water Manag..

[44] Mulovhedzi N.E., Araya N.A., Mengistu M.G., Fessehazion M.K., Du Plooy C.P., Araya H.T., Van der Laan M. (2020). Estimating evapotranspiration and determining crop coefficients of irrigated sweet potato (*Ipomoea batatas*) grown in a semi-arid climate. Agric. Water Manag..

[45] Shammout M.A.W., Qtaishat T., Rawabdeh H., Shatanawi M. (2018). Improving Water Use Efficiency under Deficit Irrigation in the Jordan Valley. Sustainability.

[46] Yang H., Liu H., Zheng J., Huang Q. (2018). Effects of regulated deficit irrigation on yield and water productivity of chili pepper (*Capsicum annuum* L.) in the arid environment of Northwest China. Irrig. Sci..

[47] Rykaczewska K. (2013). The Impact of High Temperature during Growing Season on Potato Cultivars with Different Response to Environmental Stresses. Am. J. Plant Sci..

[48] Walworth J.L., Carling D.E. (2002). Tuber initiation and development in irrigated and non-irrigated potatoes. Am. J. Potato Res..

[49] Allen R.G., Pereira L.S., Raes D., Smith M. (1998). Crop Evapotranspiration-Guidelines for Computing Crop Water Requirements.

